# Advances in diagnostic assays for *Clostridioides difficile* infection in adults

**DOI:** 10.3389/fcimb.2024.1492511

**Published:** 2024-12-10

**Authors:** Dong-ang Liu, Shiyu Chen, Ruiyao Hu, Yuting Qiu, Keyi Chen, Yue Xu, Jinghua Yuan, Xinling Zhang, Xiaoping Li

**Affiliations:** ^1^ Key Laboratory of Pollution Exposure and Health Intervention of Zhejiang Province, Shulan International Medical College, Zhejiang Shuren University, Hangzhou, China; ^2^ Key Laboratory of Artificial Organs and Computational Medicine in Zhejiang Province, Shulan International Medical College, Zhejiang Shuren University, Hangzhou, China

**Keywords:** *Clostridioides difficile* infection, adults, diagnostic assays, CRISPR, nucleic acid amplification tests, gene sequencing technology

## Abstract

*Clostridioides difficile* (*C. difficile*) was a gram-positive anaerobic *bacterium* in the gut, exhibiting clinical manifestations ranging from mild diarrhoea to fatal pseudomembranous colitis. *C. difficile* infection (CDI) remains a serious public health problem and accounted for an estimated 360,075 cases in the United States in 2021. It has attracted the utmost attention of the world health organization (WHO). Since publication of a review of the diagnosis of CDI in adults, new clinical diagnostic assays have become available and clinical practice guidelines were updated. This paper presents a comprehensive review of contemporary laboratory diagnostic approaches for CDI in adult patients, with a focus on the utilisation and potential advancements of five sophisticated methodologies, CRISPR in conjunction with nucleic acid amplification tests (NAATs), gene sequencing technology, ultra-high performance liquid chromatography-mass spectrometry, Raman spectroscopy, and real-time cell analysis (RTCA). It can provide new perspectives and ideas for the early diagnosis of CDI in clinical settings.

## 1 Introduction

**Figure 1:**
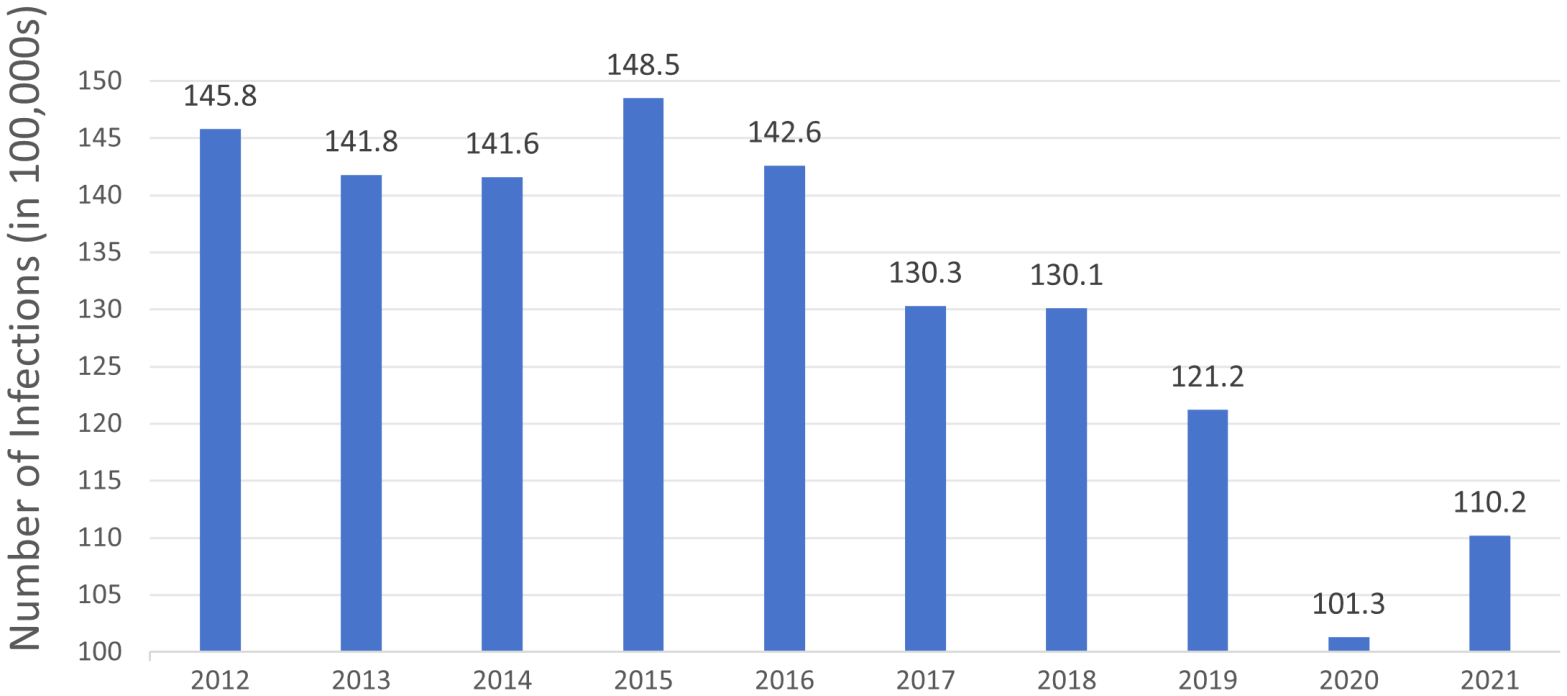
The number of *Clostridioides difficile* infection cases in North America from 2012 to 2021 over a decade. The data is derived from the Centers for Disease Control and Prevention (CDC) Annual Reports for the Emerging Infections Program for *Clostridioides difficile* Infection from 2012 to 2021.

*C. difficile* was a spore-producing anaerobic *bacterium* that has become a major pathogen causing hospital-acquired infections (HAIs) and community-acquired infections (CAIs) worldwide, especially in North America (**Figure 1**) (Feuerstadt et al., 2023; Okafor et al., 2023). Once antibiotic treatment disrupted the normal intestinal flora, *C. difficile* colonised the colon and induced diarrhoea and pseudomembranous colitis (McDonald and Lee, 2015; Theriot and Young, 2015). The global burden of CDI was exacerbated by the emergence of highly virulent and antibiotic-resistant strains (Åkerlund et al., 2008; Kuijper et al., 2006; Moore et al., 2013). A report from the Centers for Disease Control and Prevention (CDC) 2021 emphasised the persistent risk of CDI infection to patient health (National Center for Emerging and Zoonotic Infectious Diseases (U.S.). Division of Healthcare Quality Promotion. 2023). Women and Caucasians were at higher risk of developing CDI infection, which increased with age (Guh et al., 2020). To accommodate evolving clinical requirements and epidemiological traits, Conventional diagnostic assays, including toxigenic culture (TC) and cytotoxicity detection (CCTA) (Planche and Wilcox, 2011), were time-intensive and inefficient in rapidly differentiating between toxigenic and non-toxigenic strains. It could not meet the current requirements of high volume, high speed and high accuracy. With the innovation of molecular technology, quantitative polymerase chain reaction (qPCR) and loop-mediated isothermal amplification (LAMP) technologies were used by NAATs to rapidly amplify and detect target nucleic acids (El-Daly, 2024). As a result, CDI could be diagnosed earlier, with greater sensitivity, specificity, and accuracy, which allowed effective treatment strategies to be implemented more quickly and efficiently. Meanwhile, *C. difficile* mainly produces three exotoxins, including TcdA, TcdB and CDT, of which TcdB is the key pathogenic factor (Alam and Madan, 2024; Luo et al., 2022). Based on sequence variations, TcdB had been classified into several subtypes, mainly including TcdB1, TcdB2, TcdB3, and TcdB4. There were significant differences in receptor recognition and pathological effects among different TcdB subtypes, with TcdB1 being able to utilize Chondroitin Sulfate Proteoglycan 4 (CSPG4) and Frizzled (FZD), TcdB2 relying predominantly on CSPG4, TcdB3 favouring FZD, and TcdB4 not relying on these known receptors (Pan et al., 2021). This difference in receptor preference directly affected the severity of infection and pathological changes. Mouse models showed that TcdB1 caused the most severe pathological effects, whereas TcdB4 caused the least, further validating the significant difference in disease progression between the two subtypes. Thus, detection of different toxin subtypes of CDI not only helped to identify the infection, but also provided important information about the severity of infection and disease progression, which was crucial for clinicians to choose the appropriate therapeutic regimen. And complex molecular technologies including next-generation sequencing (NGS) and nanopore sequencing (NS). Not only could it better understand the epidemiology and molecular mechanisms of CDI (Goodwin et al., 2016), but we might also effectively differentiate between toxin types. In settings with limited resources, CRISPR-Cas was known for its fast and sensitive detection (Yang, 2022; Jiang et al., 2023a). Ultra-high performance liquid chromatograph-mass spectrometry (UPLC-MS) and Raman spectroscopy (RS) have also been introduced into CDI diagnosis (Mullish et al., 2022; Malyshev et al., 2023; Koya et al., 2018). Infection-specific biomarkers could be rapidly identified by metabolic profiling and molecular vibration analysis. In transitioning from conventional diagnostic assays to advanced diagnostic assays, these technological advancements aimed to improve CDI diagnosis accuracy and clinical response times. Each new approach has built on the advantages of previous approaches, it made up for some limitations. This paper can offer valuable perspectives for clinical practitioners, researchers, and public health policymakers to refine strategies for diagnosing and managing CDI. It is designed to offer valuable perspectives for clinical practitioners, researchers, and public health policymakers to refine strategies for diagnosing and managing CDI.

## 2 Traditional laboratory diagnostic assays for *C. difficile*

### 2.1 Microbiological culture

#### 2.1.1 *Clostridioides difficile* anaerobic culture

*C. difficile* was a specialised anaerobic *bacterium*, with strict requirements for culture conditions, it was not easy to grow under conventional anaerobic culture conditions. When anaerobically cultured in cycloserin-cefoxitin-fructose agar (CCFA) medium for 24-48 h, it could form white or yellowish colonies with irregular edges and typical horse faeces odour, and yellow-green fluorescence might be seen under ultraviolet irradiation (Zhou Yong et al., 2024). After the culture of CCFA, it could be identified according to the morphology of the colonies, fluorescence characteristics, odour and the results of Gram staining, and the majority of the tests could be completed in 24 hours, and the sensitivities of its detection at 24 hours and 48 hours could be up to 91.9%, 97.3%, and the sensitivity of its detection at 24 hours and 48 hours was up to 97.3% (Yang et al., 2014). Therefore, *C. difficile* isolation and culture, as a traditional assay, which could provide a more comprehensive understanding of its epidemiological characteristics and provide a basis for clinical treatment due to its high sensitivity of 94%-100% and high specificity of 99% (Napolitano and Edmiston, 2017), and the availability of the corresponding strains for molecular typing and drug-resistance analysis. It was mostly used as a gold standard for comparison of new detection assays, evaluation of new treatment assays and monitoring of clinical drug resistance. However, its shortcomings should not be ignored. Separation and culture took a long time, and it could not clearly distinguish between virulent and non-virulent strains, thus providing no basis for rapid clinical diagnosis.

#### 2.1.2 Cytotoxicity assays

*C. difficile* is divided into toxin-producing strains and non-toxin-producing strains, and its identification relies on CCTA (Antonara and Leber, 2016). Fresh patient faeces were diluted, centrifuged, and incubated with Vero or HepG2 cells with or without antitoxin A/B-neutralising antibodies for 24-48 hours. TcdA and TcdB were determined by detecting the cytopathic effect (CPE). Confirmation of *C. difficile* in the sample as a toxin-producing strain if CPE occurs. CCTA was considered the gold standard of traditional laboratory reference assays for confirming the diagnosis of CDI (Planche and Wilcox, 2011). However, the sensitivity of TC was generally considered to be between 75% and 85%, which was a highly sensitive and specific test. While it could identify CDI presence in samples, its time-consuming and labour-intensive nature increased the risk of contamination during complex sampling, culturing, and experimental procedures (Burnham and Carroll, 2013). In addition, CCTA was not suitable for immediate diagnosis in the laboratory due to instrumentation and laboratory space requirements.

#### 2.1.3 Toxigenic culture

The TC test consists of a two-part *C. difficile* culture and a toxin-producing assay.TC is performed only when the culture result is positive, and is usually initially confirmed by adding a drop of treated faecal filtrate to a monolayer of human foreskin fibroblasts and to other monolayers of human fibroblasts on a microtitre plate, and observing for the presence of CPE to initially confirm toxigenic *C. difficile* (Strachan et al., 2013). Studies have shown that the sensitivity of toxin-producing tests usually ranges from 75% to 90%, while the specificity ranges from 80% to 100%. TC has high sensitivity and specificity, but because TC only detects the *in vitro* toxin-producing ability of *C. difficile*, it cannot determine toxin production by *C. difficile in vivo*, and it cannot differentiate whether the toxin-producing *C. difficile* is causing a true infection *in vivo*. Similar to CCTA, TC has two major disadvantages of being time-consuming and labour-intensive, and is not conducive to immediate diagnosis; therefore, TC is mainly one of the gold standards of traditional laboratory reference assays for confirming CDI (Planche and Wilcox, 2011). It is generally not applicable to clinical laboratory diagnosis.

### 2.2 Immunologic tests

#### 2.2.1 Glutamate dehydrogenase

The antigenic protein GDH is widespread and highly expressed on the surface of all *C. difficile* strains. In patient samples, GDH is detected using the GDH assay to make a diagnosis. The GDH assay is a brief, cost-effective test with high sensitivity and a strong negative predictive value (NPV) (Shetty et al., 2011). There is a 90-100% sensitivity, a negative predictive value of more than 99%, and a negative GDH result can be considered negative. CDI can be excluded if the test is negative for GDH, while positive tests for toxins or toxin genes are required to confirm the test, and are often used in conjunction with enzyme immunoassay (EIA) (Zangiabadian et al., 2023), GeneXpert real-time PCR (RT-PCR) (Kameli et al., 2024), and so on. However, because both toxin-producing strains and non-toxin-producing strains produce GDH, and other strains can also produce GDH, its specificity is low, so it cannot be applied solely to laboratory diagnostic tests. As a result, it is often used as a preliminary screening test for CDI.

#### 2.2.2 Enzyme immunoassay

Using antibodies coupled to enzymes, the EIA detects specific biomarkers by binding antigens and converting the substrate into an observable product. Traditionally, GDH, the exotoxins TcdA and TcdB have been used to achieve laboratory diagnosis. The GDH EIA demonstrated a sensitivity of 82% (95% CI: 79-84) and a specificity of 91% (95% CI: 90-92), while the Tox A/B EIA showed a sensitivity of 75% (95% CI: 70-79) and a specificity of 95% (95% CI: 94-96) (Zangiabadian et al., 2023). EIA includes ELISA and immunochromatographic assay. Ramakrishnan et al. used the Duoset ELISA kit (R&D Systems) to assess neutrophil elastase and lipid transporter protein-2 levels in plasma samples (Ramakrishnan et al., 2024). Neutrophil elastase and lipid transporter-2 levels in patient plasma were determined at a 1:120 and 1:100 ratio, respectively. By comparing ELISA results from patients with CDI and healthy controls, the researchers found that plasma levels of neutrophil elastase and lipid transfer protein-2 were significantly elevated in patients with acute CDI. As faecal levels of calreticulin (FC) and lactoferrin (FL) may be associated with the severity and risk of recurrence of CDI (Gallo et al., 2018; Kim et al., 2017). Fernández et al. applied EIA in order to help predict recurrence and severity of CDI (Ágreda Fernández, 2024). They extracted faecal samples from patients using a CALEX^®^ bottle cap extraction device, and on the one hand, FC levels were measured by an FDA-approved commercial ELISA kit (Bühlmann fCAL^®^ ELISA) (Hamer et al., 2022). On the other hand, stool FL levels were measured by a commercial EIA kit (DRG EIA6038) and extracted using the EIA-5674 sample preparation system. The FC and FL prediction study results for CDI were then compared between different threshold values to determine diagnostic accuracy. The Youden Index was used to determine the optimal cut-off values to maximise the overall validity of the CDI diagnostic test (Youden, 1950). Among its advantages, EIA has the advantage of being simple and highly specific for distinguishing between toxin-producing and non-toxin-producing *C. difficile* strains. The disadvantage is that time-consuming, the sensitivity fluctuates greatly and false-negative results are easy to occur. Its positive predictive value (PPV) is low, resulting in omission of diagnosis for patients with mild disease, and strains cannot be obtained for further research, such as genotyping. The existing studies suggest it cannot be used separately as a diagnosis of CDI.

### 2.3 Nucleic acid amplification tests

#### 2.3.1 Quantitative polymerase chain reaction

qPCR is a molecular diagnostic technique that allows for the continuous monitoring of DNA amplification, with data acquisition occurring in sync with each cycle of the amplification process. This assay is crucial for correlating the initial copy number of the target nucleic acid with the rate of fluorescence increase, where a higher copy number is indicative of an earlier and more pronounced rise in fluorescence. The sensitivity of qPCR is paramount, as it enables the detection of even minute quantities of nucleic acid, which is particularly relevant for the detection of CDI. The most commonly used product assays for the qPCR process are SYBR Green and hydrolysed probes (Busi, 2020). Adrianne C Maestri et al. compared two qPCR diagnostic assays targeting the CDI *tcdB* gene (Maestri, 2022). Firstly, primers were designed using Primer3 software and *C. difficile* 630 (Costa et al., 2016; Quesada-Gómez et al., 2015), and the specificity of primers was verified by MegaX software and Primer-blast (Kumar et al., 2018). Then real-time PCR designs and controls were amplified and detected using ABI 7500, and PCR reaction conditions were established, including annealing temperature and amplification cycle. Sensitivity, reproducibility, specificity, and accuracy were analysed. Finally, DNA was extracted from *C. difficile* ATCC BAA1870 bacterial suspension and tested for sensitivity and specificity at different concentrations. These two qPCR CDI assays were compared with the conventional GDH plus TC CDI assay. 97.9% sensitivity and 99.1% specificity were achieved with the SYBR Green assay, while 87.5% sensitivity and 100.0% specificity were achieved with the hydrolysis probe assay (Maestri, 2022). Both had diagnostic accuracies of 99.0% and 98.5%, respectively, and these findings underscore the ability of qPCR to reliably detect CDI in clinical samples. Xiao-xi Jia et al. designed a single-tube multiplex qPCR assay for the direct detection of toxin-producing *C. difficile* in faecal samples (Jia et al., 2023), which facilitates the diagnostic efficiency of CDI. The assay can also differentiate between multiple toxin genes and subtypes, such as *tcdA*, *tcdB*, and *cdtB* (Kubota et al., 2014; Luna et al., 2011). These genes are key for the detection of CDI. Additionally, the assay includes the internal control gene chip. It is built to work with various qPCR platforms, ensuring flexibility. The assay also integrates with the point-of-care-testing (POCT) system. This integration significantly boosts the efficiency of detection. Furthermore, it improves the sensitivity of the assay. The assay exhibits a limit of detection (Lod) from 10^1^ to 10^3^ gene copies per microliter, with coefficients of variation below 3% for both inter-batch and intra-batch analyses, signifying excellent reproducibility. In faecal samples, the limit of detection was found to range between 5×10^2^ to 5×10^5^ colony-forming units per gram (CFU/g), underscoring the robustness of the assay for CDI diagnosis. The qPCR assay exhibited a high concordance rate of 98.4% with conventional PCR and ELISA (Costa et al., 2016), demonstrating equivalent sensitivity in identifying both toxin-producing and non-toxin-producing strains of *C. difficile*. This technique offers the advantages of high specificity and rapid detection, allowing for quantitative analysis compared to traditional PCR. However, it requires the use of specific fluorescently labelled probes and specialised qPCR equipment, which can be cost-prohibitive. Additionally, the assay necessitates sophisticated experimental manipulation and data analysis, mandating the expertise of trained professionals for both operation and result interpretation.

#### 2.3.2 Loop-mediated isothermal amplification

LAMP is a nucleic acid amplification technique used to detect and quantify DNA molecules. The most important feature is that it is performed at a constant temperature, usually 60-65°C. There is no need to rely on temperature cycling equipment such as PCR, simplifying the experimental conditions. LAMP uses four specific primers to recognise six target regions, including two outward primers, two inward primers, and two loop primers. The aim is to recognise the six regions of target DNA within a short period of time (30 ~ 60 min). Using rapid amplification of nucleic acids, different parts of the target DNA are combined to produce many product DNA molecules. The colour change or turbidity is visible without the use of subsequent detection assays such as gel electrophoresis, thus enabling rapid detection of the target gene. LAMP, an assay that detected the presence of binary toxin genes, was used by Lan Yu et al. to diagnose CDI (Yu et al., 2017). For the binary toxin encoding genes of *C. difficile*, the *cdtA* and *cdtB* gene sequences were analysed using Primer Explorer Version 4 software, and eight primers for *cdtA* and six primers for *cdtB* were designed (Lin et al., 2022). The LAMP reaction was then performed using the Loopamp^®^ DNA amplification kit from Eiken Chemical Co. Ltd. The optimal reaction conditions were determined to be 60 min at 60°C based on the turbidity change data at 650 nm from a real-time turbidity monitor. The reaction system was supplemented with Loopamp^®^ Fluorescence Detection Reagent from Eiken Chemical Co. Ltd. This reagent contains a metal indicator compatible with the reaction system. Visual judgement was made by colour change (from orange to green) (Girão, 2021). Subsequently, a specificity test was carried out to test the specificity of the LAMP reaction using 25 different bacterial strains (including *C. difficile*), which showed that the LAMP reaction only reacted to *C. difficile* and was negative for all other *bacteria*. The sensitivity test, which compared the LAMP assay and traditional PCR for diagnosing CDI, found that the detection limit of LAMP is 24.8 pg/µL, which is 10 times lower than that of traditional PCR (Yu et al., 2017). Lin M. et al. developed a LAMP assay for the tetM gene in C. difficile strains cultured from faeces, which efficiently amplified the target DNA within 60 min at a constant temperature of 62°C, with a Lod of 36.1 pg/μL, representing a 100-fold increase in sensitivity over traditional PCR (Lin et al., 2022). Hiber Gene’s LAMP assay uses a novel LAMP targeting the tcdA and tcdB genes to diagnose CDI. When compared to the GDH toxin A/B test (C. diff Quik Chek^®^), Hiber Gene’s LAMP demonstrated 100% sensitivity and 95.8% specificity. When compared to the multiplexed NAATs FilmArray™ GI panel^®^ (BioFire), the sensitivity was 81.2%, the specificity was 100%, and the concordance was 96.38% (Gandelman et al., 2011). Therefore, LAMP technology offers advantages such as high specificity and selectivity, high sensitivity, fast reaction speed, and a simple operational procedure, with results that can be visualised and observed in a short time (Norén et al., 2014).

## 3 Advanced laboratory diagnostic assays for *Clostridioides difficile*

### 3.1 Next-generation sequencing

As a DNA sequencing assay, NS is also known as high-throughput sequencing (HTS). It is based on the principles of PCR and Gene Chip, which enables sequencing while synthesising. NGS technology determines the DNA sequence by capturing special markers (usually fluorescent molecules) on bases added during DNA replication. This technology is characterised by its high throughput and short read lengths. Major technology platforms include the Roche 454 FLX, Illumina MiSeq, and Illumina HiSeq, etc. NGS is particularly suited for amplicon sequencing, such as 16S, 18S rRNA genes and variable regions of internal transcribed spacers (ITS). For sequencing genomic and macrogenomic DNA, Metagenomic Shotgun Sequencing (MSS) is required (Falony et al., 2019; Vasilescu et al., 2022), which involves breaking DNA into small fragments (e.g., 30bp) for sequencing (Quince et al., 2017). In a study to identify diarrhoea-associated pathogens in faecal specimens. Two sequencing technologies using NGS platforms were compared. Sequencing of the 16S rRNA gene started with total nucleic acids from *C. difficile* extracted from faecal samples for prior sample processing, followed by PCR amplification of the V3 to V5 regions of the 16S rRNA gene using specific primers (357F and 926R). The amplicons were then purified as well as mixed equimolarly. Finally, pyrophosphate sequencing was performed on a Roche 454 Titanium platform. Low-quality reads (average mass <35), short reads (<200 bp), and reads containing sequences from the chimeric 16S rRNA gene were removed, while high-quality sequences were classified from phylum to genus level by the Ribosomal Database Project Naive Bayesian Classifier. Of the 22 C*. difficile*-positive samples, 20 (90.9%) were detected by 16S rRNA gene sequencing (Woo et al., 2005; Zhou et al., 2016). MSS first performed sample processing to convert total *C. difficile* nucleic acids to DNA and construct single-indexed sequencing libraries. The DNA is then interrupted using a Covaris instrument and subjected to end repair, A-tail addition, junction ligation and PCR amplification. Finally, MSS was performed on the Illumina HiSeq platform to generate 100 bp paired-end reads. Reads were mass trimmed, host contamination removed and low complexity regions masked. Approximately 5000 reference genomes were aligned using RTG mapping to identify bacterial and fungal species. MSS was detected in 86.3% of *C. difficile*-positive samples detected (Zhou et al., 2016). Both assays have their advantages and limitations. 16S rRNA gene sequencing is less costly but may have PCR-related bias and limited classification accuracy. MSS, on the other hand, is more costly and has a huge amount of data, requiring large computational resources for data processing and storage. These two assays can complement each other to improve the diagnostic accuracy of CDI. Whole Genome Sequencing (WGS) is one of the most widespread applications of NGS technology (Goodwin et al., 2016). It specifically refers to the process of sequencing the entire genome, focusing on obtaining sequence information about the entire genome. WGS can obtain complete genomic information about a *C. difficile* strain infecting a patient by sequencing the entire genome of the strain. This information encompasses the strain genome sequence, the proteins encoded by the genes, and data regarding other related genes (Capobianchi et al., 2013; Miller et al., 2013). Study by Helena et al., the WGS technique was able to provide more detailed and precise information about the strain compared to traditional PCR-ribotyping (Seth-Smith et al., 2021). Single nucleotide polymorphisms (SNPs) can be detected by WGS, which is particularly useful in tracing the source of infection and the chain of transmission between cases (Kuenzli et al., 2020). In contrast, PCR-ribotyping, although commonly used and rapid, has limited resolution and does not provide the same detailed genetic information. Meanwhile, WGS analysis enables the tracking of genetic variation in *C. difficile* and the identification of affinities between different strains, which can lead to inferences about the source of infection and the path of transmission (Goyal et al., 2020). A Canadian study utilised whole genome sequencing to identify genetic and epidemiological correlations between CDI cases and previously infected or colonised individuals, highlighting the role of colonised patients in transmission. Laboratory diagnosis of C. difficile strains was conducted using WGS (Hargreaves et al., 2016). By comparing genomic sequence differences between *C. difficile* strains, CDI can be identified and targeted therapeutic measures can be implemented more accurately.

### 3.2 Nanopore sequencing

NS is also called ab initio sequencing technology, i.e., single-molecule real-time DNA sequencing. NS is represented by PacBio’s single molecule real time sequencing (SMRT) and Oxford nanopore technologies (ONT). Their greatest advantage over the previous two generations is single-molecule sequencing, which does not require PCR amplification. Conventional short-read-length sequencing techniques tend to lead to fragmentation of genome sequences, especially when dealing with *bacteria* with complex genome structures and repetitive sequences. In contrast, SMRT sequencing can generate high-quality complete genome sequences, which is particularly important for pathogens such as *C. difficile*. Through SMRT, the researchers identified genomic differences and rearrangements between different isolates of *C. difficile* (Hargreaves et al., 2016). These differences include insertions and deletions of mobile genetic elements (MGEs), such as the differential presence of the Tn6164 and Tn6293 transposons in different strains. These MGEs not only affect the structure of the genome but may also transfer genes between strains through horizontal gene transfer mechanisms. In addition, SMRT was able to detect the presence of methylation modifications. An enzyme called *C. difficile* adenine methyltransferase a (CamA) has also been found to play an important role in *C. difficile* reproduction (Oliveira, 2019), especially during spore formation. When the DNA methylation corresponding to CamA was lost, *C. difficile* spore formation was reduced by about half. This suggests that detecting the DNA methylation status of C. difficile could serve as a potential indicator for diagnosing CDI, which is crucial for understanding the regulation of bacterial gene expression and adaptation to different environments. In contrast to 16S rRNA gene sequencing, which is limited in revealing the structure of microbial communities due to the taxonomic resolution is restricted to the genus or family level. PacBio’s SMRT, which is capable of generating very long read lengths, avoids primer bias and therefore has a significant advantage in terms of resolution and accuracy. Sadowsky et al. demonstrated that microbial communities can be analysed more efficiently with SMRT, making the identification of *C. difficile* more accurate (Sadowsky et al., 2017). The role of nanopore electrical signal sequencing in CDI is to analyse in detail the species and strains of the donor gut microbiota and how they colonize and affect the recipient in a high-resolution manner. This technique enables rapid sequencing of bacterial ribosomal RNA gene clusters, including the 16S-ITS-23S region, to accurately identify strains of *C. difficile*. In this way, researchers such as Benítez-Páez can track the transfer process of specific *bacteria* such as *C. difficile* (Benítez-Páez et al., 2022).

### 3.3 Clustered regularly interspaced short palindromic repeats/CRISPR-associated

**Figure 2:**
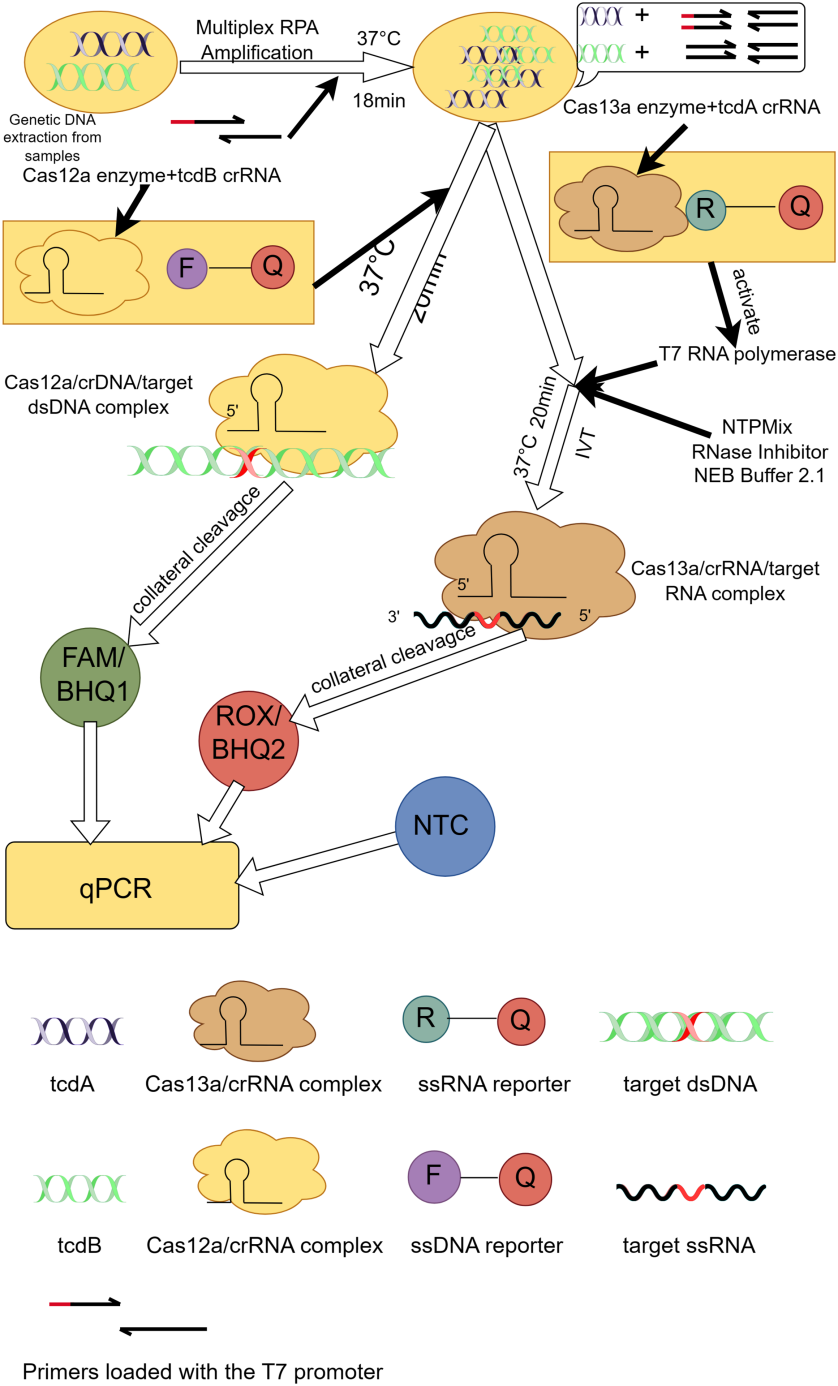
Principle of the OC-MAB platform. DNA samples of *C. difficile* were isothermally amplified in multiplex RPA, *tcdA*, *tcdB* were subjected to CRISPR dual-system cleavage, and the cleavage signals and NTC negative controls were compared with the results by fluorescence. This figure was drawn by Figdraw.

The evolution of CRISPR-Cas technology in diagnostics demonstrates its diverse applications as a revolutionary molecular biology tool (Li et al., 2023a). Initially, the CRISPR-Cas system was discovered as a natural immune system for *bacteria* and archaea and attracted extensive interest from the scientific community. Subsequently, researchers began to explore its potential in gene editing and molecular biology. Important advances in the use of CRISPR-Cas technology for diagnostic applications date back to 2015, when Zetsche et al. first described Cas12a (formerly Cpf1), a single RNA-guided nucleic acid endonuclease capable of cleaving double-stranded DNA in cis (Zetsche et al., 2015). Subsequently, Chen et al. found that Cas12a also demonstrated the ability to cut single-stranded DNA in trans, which laid the foundation for the development of novel nucleic acid detection platforms (Chen et al., 2018). Building on this discovery, platforms such as DNA nucleic acid endonuclease targeting CRISPR trans reporter genes (DETECTR) and the 1-hour low-cost versatile and efficient system (HOLMES) have been developed, combining Cas12a with recombinase polymerase amplification (RPA) for rapid detection of various pathogens (Chen et al., 2018; Li et al., 2018). On the other hand, breakthroughs in specific nucleic acid detection were triggered by the discovery of Cas13a (earlier known as C2c2), which exhibited trans-cleavage activity against RNA, leading to the development of the specific highly sensitive enzyme reporter gene unlocking (SHERLOCK) platform (Gootenberg et al., 2017). The platform utilized the non-specific cleavage activity of Cas13a on single-stranded RNA (ssRNA) in combination with fluorescent or other indicators to enable rapid detection of a wide range of pathogens, including SARS-CoV-2 (Li et al., 2023b, He et al., 2023). With further technological evolution, the introduction of orthogonal CRISPR strategies further enhanced the possibility of multiplexed detection, and Gootenberg et al. introduced the SHERLOCK platform (Gootenberg et al., 2018), which, for the first time, utilised an orthogonal CRISPR system to achieve simultaneous detection of multiple targets in a single reaction. This advancement not only expanded the range of detection, but also improved the sensitivity and specificity of the assay. Tian et al. further extended this concept by developing a portable fluorescent detection device for rapid detection of pathogens such as SARS-CoV-2 in a single tube, which greatly facilitated the application and popularization of immediate diagnostic technologies (Tian et al., 2022). Tong Jiang et al. took it a step further by investigating the development of a new diagnostic platform for laboratory diagnosis of CDI called orthogonal CRISPR system combined with multiplex recombinase polymerase amplification (OC-MAB) (**Figure 2**) (Jiang et al., 2023b), which combines two CRISPR systems (Cas12a and Cas13a) and multiplex recombinase polymerase amplification (RPA) technology designed to simultaneously recognize the toxin genes of *C. difficile tcdA* and *tcdB*. *C. difficile* DNA was first extracted from the samples. specific primers were then designed to target the *C. difficile* toxin genes, *tcdA* and *tcdB*, and the target DNA sequences were isothermally amplified using RPA technology (Kaminski et al., 2021). T7 RNA polymerase was then used to transcribe *tcdA* into single-stranded RNA (ssRNA), while *tcdB* was left intact as double-stranded DNA (dsDNA). Next, the Cas12a and Cas13a proteins were specifically recognised by DNA and RNA, respectively, and their non-specific cleavage activity was activated (Chen et al., 2018; Myhrvold et al., 2018). This leads to cleavage of the labelled DNA and RNA probes, releasing a fluorescent signal. On the one hand, the fluorescence signal after cleavage can be detected using the dual-channel fluorescence detection system of the qPCR instrument. And the presence or absence and intensity of the fluorescent signal can be used to determine whether the *C. difficile* toxin gene is present in the sample. On the other hand, the cutting product of the CRISPR-Cas system can be combined with immunochromatographic test strips labelled with antibodies for visual detection. A colour change is displayed on the test strip, allowing a visual determination of the presence or absence of the *C. difficile* toxin gene in the sample. The OC-MAB platform not only excels in a laboratory setting but also has the potential to be used in resource-poor areas or where rapid detection is required. It has an extremely low detection limit of 10^2^ to 10^1^ copies/mL with excellent sensitivity and specificity (Jiang et al., 2023b). The OC-MAB platform demonstrates comparable or superior performance to traditional qPCR in detecting *C. difficile*.

### 3.4 Ultra-performance liquid chromatography-mass spectrometry

The coupling of high-performance liquid chromatography (HPLC) and mass spectrometry (MS) has significantly improved the separation capability and sensitivity of sample analysis. It enables rapid and accurate detection and identification of components in complex mixtures, providing a wealth of data and quantitative analysis capabilities for research. UPLC-MS has proven invaluable for diagnosing CDI by providing a comprehensive analysis of the metabolome in faecal samples (Zhou et al., 2018). UHPLC-MS was chromatographically separated using a Waters ACQUITY UPLC system and a BEH C18 analytical column (Rathod et al., 2019). Separation efficiency and selectivity were improved by mobile phase A (water/formic acid) and mobile phase B (acetonitrile/formic acid) (Cao et al., 2011; Huang et al., 2013). A linear gradient elution procedure was used for faecal sample analysis. Mass spectrometry analysis was then performed in positive ion electrospray mode. Peak intensities in the raw UHPLC-MS data were detected, integrated, and normalised using MarkerLynx Applications Manager software (MarkerLynx). Pattern recognition analysis was performed using principal component analysis (PCA) and partial least squares discriminant analysis (PLS-DA) (Mbunge et al., 2022; Yahaya et al., 2016). Linear discriminant analysis (LDA) based on sequential feature selection was performed using Matlab software to diagnose the cause of diarrhoea. Finally, the Human Metabolome Database and PubChem Compound Database were searched and mass spectra and retention times of potential biomarkers were compared with authentic standards for identification. The *C. difficile*-positive, *C. difficile*-negative, and healthy control groups showed significant clustering differences in the PCA score plots. In the PLS-DA model, the *C. difficile*-positive group showed a clearer separation from the *C. difficile*-negative group and the healthy controls. A total of eight classes (13 types) of markers were identified, including fatty amides, sphingomyelins, bile acids, amino acids, carnitine, lecithin (LPC), and esters. An LDA model based on capsiamide, dihydrosphingosine, and glycochenodeoxycholic acid was developed for the identification of CDI in diarrhoea (Dawkins et al., 2022). The model showed leave-one-out cross-validated CDI diagnosis (LOOCV) accuracy, sensitivity, and specificity of the training set of 90.00%, 93.33%, and 86.67% respectively. While the predicted accuracy, sensitivity, and specificity of CDI diagnosis for the external validation set were 78.57%, 83.33%, and 75.00% respectively (Mbunge et al., 2022). The area under the receiver operating characteristic curve (AUC) was 0.9 and 0.7917 for the training and validation sets, respectively. *C. difficile* diarrhoea has a unique faecal metabolomic profile compared to other hospital-onset diarrhoea. The UPLC-MS metabolomics model is expected to be a useful tool for diagnosing C. difficile diarrhoea (Weingarden et al., 2014). The disadvantages of UHPLC-MS technology include high cost, complexity of operation and data processing, low throughput, and limitations for specific analytes, which limit its widespread use.

### 3.5 Raman spectroscopy

Raman spectroscopy is an analytical spectroscopy technique that utilizes the Raman effect to study the molecular vibration and rotation states of matter (Koya et al., 2019). In Raman spectroscopy, a sample is excited by monochromatic light (usually a laser) and scatters. When the laser beam interacts with the molecules in the sample, every 106 to 108 photons undergo elastic scattering (Rayleigh scattering, where there is no change in the energy of the scattered versus incident photons), while about 1 photon undergoes inelastic scattering (Raman scattering, which involves a change in the energy of the scattered versus incident photons) (Hassanain et al., 2021). Hassanain et al. developed a novel laboratory diagnostic tool based on surface-enhanced Raman scattering (SERS)-based lateral flow assay (LFA). This technique uses gold nanoparticles (AuNPs) as an enhancement substrate for SERS signals. Specific antibodies and Raman reporter molecules (e.g. malachite green isothiocyanate, MGITC) were bound to the surface of AuNPs by physical adsorption to form SERS nanotags. These nanotags specifically recognise and bind to target biomarkers (SlpA and TcdB) (Hassanain et al., 2021). The capture antibodies were then sprayed at specific locations on the nitrocellulose membrane to form test and control lines. The test line is used to capture specific biomarkers while the control line is used to confirm the functionality of the test strip. The SERS nanolabels are mixed with the sample such that the antibodies on the nanolabels form immune complexes with the biomarkers in the sample (Wang et al., 2024). Two separate test lines, corresponding to SlpA and TcdB, were designed on a lateral flow test strip. The formed immune complex solution was added dropwise to the lateral flow test strip, and capillary action was used to cause the solution to migrate along the test strip. When the immune complex reaches the test line, it binds to the pre-immobilised capture antibody and forms a ‘sandwich’ structure, resulting in a change in the colour of the test line, thus enabling the visualisation of the biomarker. A handheld Raman spectrometer can be used to scan the test line and further collect Raman signals generated by the SERS nanotag (Joung et al., 2022). The concentration of biomarkers can be quantified by analysing the intensity changes of specific Raman peaks. Compared to traditional assays such as ELISA, SERS-based LFA not only offers higher sensitivity (minimum observed concentration of 0.01 pg/μL) and specificity, but also completes the assay in a shorter period of time (only 20 minutes) using dual detection and flow measurement test strips. The platform can be used in the field with a handheld Raman spectrometer suitable for use at the point-of-care (POC), facilitating rapid detection in the field and reducing sample handling and analysis time, enabling faster diagnosis and management of CDI cases (Hassanain et al., 2021). However, RS still requires some technical knowledge and equipment support.

### 3.6 Real-time cell analysis

Real-time Cell Analysis (RTCA) was a marker-free, non-invasive assay for monitoring cell status. Cell growth and response to toxins were assessed by detecting changes in electrical impedance on cell culture plates (Solly et al., 2004). RTCA quantifies TcdB toxin concentration by changes in cell index (CI) associated with changes in electrical impedance, thereby reflecting changes in the number, morphology, and degree of adhesion of toxin cells. In 2010, Ryder et al. monitored cytotoxic effects using the hypersensitive nature of HS27 cells to TcdB toxin in the RTCA platform. By using the platform it was learned that the Lod was 0.2 ng/mL, the specificity was 99.6%, and the sensitivity was 87.5%. Based on these results, the RTCA system might be used to determine clinical CDI severity and to monitor therapeutic efficacy using toxin concentration measurements (Ryder et al., 2010). Subsequently, Huang et al. combined the immunomagnetic bead separation and enrichment technique with an improved RTCA system (RTCA version 2) in 2014. Not only was the sensitivity increased to 96.2%, but also the Lod was reduced to 0.12 ng/mL. The test results of those samples could be obtained within 24 hours (Huang et al., 2014). By 2024, Shen et al. assessed the sensitivity to TcdB using four cell lines and selected the HS27 cell line as the target cell line for testing TcdB. Compared with RTCA, neither ELISA nor PCR had high resolution. RTCA could effectively distinguish CDI from *C. difficile* colonisation (CDC) through quantifying functional and toxical TcdB (Shen et al., 2024). Therefore, RTCA could be used as an effective complementary diagnostic tool for ELISA and PCR. However, this technology faced challenges such as operational complexity, high cost, and the possibility of inhibitors in the sample that affected the accuracy of the determination.

## 4 Discussion

Laboratory diagnosis of CDI is a multistep process involving a variety of traditional and advanced techniques. Traditional assays, such as TC and CCTA, are time-consuming and do not allow rapid differentiation between virulence-producing and non-virulence-producing strains, despite their high specificity (Luo et al., 2022). In contrast, anaerobic incubation in CCFA medium can detect *C. difficile* within 24 to 48 hours, with sensitivities of up to 91.9% at 24 hours and 97.3% at 48 hours, respectively (J. J. Yang et al., 2014). However, this assay cannot provide a basis for rapid clinical diagnosis, limiting its application in immediate diagnosis. Immunological tests, although simple and time-consuming, have low specificity and are usually used as a primary screening tool. The sensitivity of the GDH test is 90% to 100%, with a negative predictive value of more than 99%, and advances in GDH-EIA allow for rapid real-time diagnosis of CDI (101). NAATs, especially qPCR and LAMP, have significantly improved the sensitivity and specificity of CDI. The development of qPCR technology demonstrates high sensitivity and specificity; the SYBR Green assay has a sensitivity of 97.9% and specificity of 99.1%, and the hydrolysed probe assay has a sensitivity of 87.5% and specificity of 100.0% and can be combined with automated molecular assays to achieve faster and more sensitive diagnostics, such as the GeneXpert system (Makristathis et al., 2017). LAMP technology has shown great potential for immediate detection with its fast, simple process and visualisation of results, and is up to 10 times more sensitive than traditional PCR. Advanced diagnostic assays, with NGS observing significant concordance across typing assays, offer advantages that have transformed epidemiologic studies, enabling high-resolution genomic analysis of *C. difficile* (Maestri et al., 2023). By contrast, NS Technology uses ONT sequencing to detect CDI of sequence type (ST)2 as part of its recent prospective genomic surveillance system using multilocus sequence type (MLST) (**Table 1**), which provides in-depth genomic information relating to *C. difficile* epidemiology and molecular mechanisms of infection (Abad-Fau et al., 2023). RTCA demonstrated significant advantages in this study. It is able to quantify functional and virulent *C. difficile* TcdB in clinical samples by monitoring changes in CI in real time. Compared to traditional assays such as ELISA and PCR, RTCA can distinguish CDI from CDC more accurately (98). Because it directly measures the biological activity of the toxin and not just the presence of toxin genes or proteins. This assay is not only rapid but also highly sensitive and specific, providing an effective clinical tool for better diagnosis and management of CDI. CRISPR-Cas technology, with its rapidity and sensitivity, offers a new strategy for the diagnosis of CDI, especially in resource-constrained settings (Bloomfield et al., 2024). Technologies such as UPLC-MS and RS provide a new dimension to the diagnosis of CDI by analysing the metabolome and molecular vibrations of samples. These technologies enable rapid identification of infection-specific biomarkers that contribute to early diagnosis and treatment, further improving diagnostic accuracy and clinical responsiveness (**Table 2**). In summary, the development of novel diagnostic platforms offers new possibilities for rapid and accurate diagnosis of CDI. Future research should focus on improving the accuracy, accessibility, and cost-effectiveness of detection technologies to better serve clinical needs.

**Table 1:** Comparison Table of NGS and NS.

Technology Type	Sequencing Principle	Read Length Features	Throughput Characteristics	Sequencing Accuracy	Sample Enrichment	Cost	Advantages	Limitations	CDI Research Applications	References
NGS	Sequencing by synthesis with reversible termination	Short read lengths, suitable for amplicon sequencing	High throughput, suitable for large-scale sample analysis	Lower error rates, requires reference sequences	Requires PCR amplification	Higher than PCR, lower than NS	High throughput, low cost, precise short read lengths	Limitations of short read lengths, requires reference sequences	Pathogen identification, 16S rRNA gene sequencing	(Goodwin, et al., 2016)
NS	Single-molecule real-time sequencing, no PCR required	Long read lengths, suitable for complex genomes	Medium to low throughput, suitable for long sequence analysis	Higher error rates, correctable by coverage	Direct sequencing, no PCR required	Particularly high	Long read lengths, no PCR required, complex genome resolution	High error rates, high costs	Genomic variation tracking, source of infection transmission chain analysis	(Hargreaves et al., 2016; Koya et al., 2019)

**Table 2:** Comparison table of traditional and advanced laboratory diagnosis of *Clostridioides difficile* infection.

Diagnostic Assay	Detected Substances	Time Required	Cost	Specificity	Sensitivity	Accessibility	Challenge level	References
*C. difficile* anaerobic culture	Colony morphology and color	1-3 days	Low	High	High	Medium accessibility, low cost of instruments, one of the gold standards	Medium challenge, time-consuming, medium personnel and operational requirements, unable to distinguish non-toxigenic strains, cannot POCT	(Zhou et al., 2024)
CCTA	Tcd B	1-3 days	High	High	Low	Medium accessibility, low cost of instruments, can distinguish between toxigenic and non-toxigenic strains, one of the gold standards	Medium challenge, time-consuming, medium personnel and operational requirements, cannot POCT	(Antonara and Leber, 2016)
TC	Toxinogenic *C. difficile*	3-5 days	High	High	Medium to high	Medium accessibility, low cost of instruments, one of the gold standards	Medium challenge, time-consuming, medium personnel and operational requirements, unable to distinguish non-toxigenic strains, cannot POCT	(Strachan et al., 2013)
GDH	GDH, TcdA and TcdB	Several hours	Low	Low	High	High accessibility, short time consumption, low cost, high NPV, high reliability, often used as an initial screening test for CDI	Low challenge, unable to distinguish between toxigenic and non-toxigenic strains	(Shetty et al., 2011)
EIA	Tcd A/TcdB	3-5 hours	Low	High	Low	High accessibility, easy to operate, can distinguish between toxigenic and non-toxigenic strains	Low challenge, low cost, time-consuming, sensitivity fluctuates greatly, prone to false negatives. Low PPV	(Zangiabadian et al., 2023)
NAAT	Toxin Genes	1-3 hours	High	High	High	High accessibility, short time, quantitative analysis possible, can implement POCT	Low challenge, high cost, medium personnel and operational requirements	(Lin et al., 2022; Maestri, 2022)
NGS	DNA sequences (*tcdA*, *tcdB*, etc.)	Several hours to one day	High	High	High	Low accessibility, high throughput, capable of metagenomic sequencing	High challenge, requires PCR amplification, high equipment cost	(Goodwin et al., 2016)
NS	DNA sequences (*tcdA*, *tcdB*, etc.), methyltransferases (CamA, etc.)	Several hours	High	High	Relatively high	Low accessibility, long read length, no PCR required, real-time sequencing	High challenge, higher error rate, relatively high equipment and time costs	(Hargreaves et al., 2016; Koya et al., 2019)
CRISPR-Cas	*tcdA*, *tcdB*	About 1 hour	Medium	High	High	High accessibility, short time consumption, high portable, suitable for POCT	Medium challenge, medium personnel and operational requirements	(Jiang et al., 2023b)
UPLC-MS	Metabolic markers (lipids, amino acids, etc.)	Several hours	High	High	Medium to high	Low accessibility, can diagnose unique fecal metabolomic features	High challenge, high cost, complex operation and data processing, low throughput, and limitations to specific analytes	(Zhou et al., 2018)
RS	Biomarkers (SlpA, TcdB, etc.)	Less than 30 minutes	Low to medium	Medium to high	High	High accessibility, short time consumption, portable, suitable for POCT	Medium challenge, medium personnel and operational requirements	(Hassanain et al., 2021)
RTCA	TcdB	Less than 24h	High	High	High	High accessibility, effective differentiation between infection and colonisation, quantitative detection of toxins	Medium challenge, operational complexity, high cost, subject to inhibitors	(Shen et al., 2024)

## 5 Conclusion

In summary, in order to effectively diagnose and treat CDI, medical laboratories have now adopted a variety of detection techniques, which include microbiological culture, immunological detection, and molecular biology detection. Microbiological culture is the most basic assay, but it takes a long time to obtain the results. Immunological detection has high sensitivity but may be interfered with by other antibodies. Molecular biology testing can quickly and accurately detect the genetic material of *C. difficile*, but it is more demanding under experimental conditions. With the continuous progress of biotechnology, new laboratory diagnostic techniques for *C. difficile* have emerged, facing different needs for different technologies, if one needs to get the test results quickly, NAATs may be a better choice. Out of the same need, new diagnostic platforms for CDI, such as RTCA, have become available. can not only diagnose CDI but can further differentiate between specific toxin-type aspects. In cases where limited resources and expertise are available, RS may be a more practical option. CRISPR technologies (Kozovska et al., 2021). With the development of CRISPR-Cas technology, the combination of isothermal amplification and CRISPR-Cas technology can rapidly detect DNA/RNA, effectively promoting the development of CRISPR-Cas technology in POCT (Mao et al., 2023). On the other hand, these technologies are indispensable tools when it comes to gene editing and in-depth molecular mechanism studies (Li et al., 2023a). These technologies will provide more options for future clinical diagnostics. However, these advanced detection technologies are not foolproof, there are still many challenges to be faced in their practical application. For example, diagnostic criteria may differ between different laboratories, which will affect the accuracy and comparability of diagnostic results. These techniques are difficult to operate and require experienced laboratory personnel. In addition, the cost of these techniques is relatively high, which restricts their popularization and application in primary healthcare institutions (Li et al., 2023b). It is only through overcoming these challenges that we will be able to continue developing, improving, and providing more timely and effective diagnostic and treatment services for patients with CDI.
